# Genetic and Clinical Characteristics of Patients With Hereditary Spherocytosis in Hubei Province of China

**DOI:** 10.3389/fgene.2020.00953

**Published:** 2020-08-18

**Authors:** Xiong Wang, Ai Zhang, Ming Huang, Li Chen, Qun Hu, Yanjun Lu, Liming Cheng

**Affiliations:** ^1^Department of Laboratory Medicine, Tongji Hospital, Tongji Medical College, Huazhong University of Science and Technology, Wuhan, China; ^2^Department of Pediatrics, Tongji Hospital, Tongji Medical College, Huazhong University of Science and Technology, Wuhan, China

**Keywords:** hereditary spherocytosis, ANK1, SPTB, variable expressivity, mutation

## Abstract

Hereditary spherocytosis (HS) is an inherited disorder characterized by anemia, splenomegaly, and spherical-shaped erythrocytes, caused by mutations in erythrocyte membrane Protein Genes (*ANK1*, *SPTB*, *SLC4A1*, *SPTA1*, and *EPB42*). We investigated molecular spectrum and genotype-phenotype correlation in HS patients in Hubei province, central China. Twenty-three patients with HS were included. A next-generation sequencing (NGS) panel targeting *ANK1*, *SPTB*, *SLC4A1*, *SPTA1*, and *EPB42* genes was used to screen potential variants. Sanger sequencing was applied to validate variants. Of the twenty-three patients, thirteen patients carried *ANK1* variants, and ten patients harbored *SPTB* variants, including ten non-sense, six indel, four splice site, one start-loss, and one missense variant. Four out of twenty-two variants in our study were known, and eighteen variants were novel. Most *ANK1* and *SPTB* variants were indel (5/12) or non-sense (7/10), respectively. Family member analysis in thirteen families showed that six variants were *de novo*. Variable expressivities were observed in a pair of twins with *ANK1* c.341C > T variant, and two unrelated patients both carried *ANK1* c.2T > A variant. Genotype-phenotype analysis found no significant difference between *ANK1* and *SPTB* regarding the levels of Hb, RBC, MCV, MCH, and MCHC. However, variants in the ANK1 death domain were associated with lower levels of MCV and MCH compared to other ANK1 domains. In conclusion, NGS is a fast way to provide a molecular HS diagnosis. We also identified unique genetic and clinical characteristics of patients with HS in Hubei Province, China. However, a large sample size is needed to further investigate the genotype-phenotype correlation.

## Introduction

Hereditary spherocytosis (HS) is an inherited disorder characterized by anemia, splenomegaly, spherical-shaped erythrocytes on blood smear, osmotically fragile spherocytes, and jaundice, and it can present with or without cholelithiasis. Its clinical manifestation varies from asymptomatic to severe and life-threatening anemia. HS is caused by mutations in various erythrocyte membrane protein genes, including ANK1 (ankyrin), SPTB (β-spectrin), SLC4A1 (Band 3), SPTA1 (α-spectrin), and *EPB42* (protein 4.2) with significant heterogeneity in the molecular deficiency (He et al., 2018). Autosomal dominant (AD) and autosomal recessive (AR) patterns of inheritance account for 75% and 25% of all the HS cases, respectively. HS prevalence varies among different racial and ethnic regions, affecting approximately 1 in 2000 individuals in northern Europe, North America, and Japan, but it is less common in African-American and southeast Asian people (Perrotta et al., 2008). The estimated prevalence is 1:100,000 in the Chinese population (Wang et al., 2015).

HS is usually diagnosed based on a positive family history, spherocytosis, jaundice, and splenomegaly. The HS phenotype may also be modified by co-occurrence with other disorders like glucose-6-phosphate dehydrogenase (G6PD) and Gilbert syndrome (Aggarwal et al., 2019; Zou et al., 2020). Application of next-generation sequencing (NGS) has led to impressive progress in genetic disorder diagnostics, and it has provided unprecedented benefits for both personalized laboratory medicine and patients with rare genetic disorders (Di Resta and Ferrari, 2018; Prodan Zitnik et al., 2018). NGS has promoted HS molecular diagnosis compared to clinical practice, including both targeted panel and whole exome sequencing (WES) (Xue et al., 2019; Qin L. et al., 2020).

Genetic and clinical features of Chinese HS cases have been frequently identified. Systematic investigation of the genotype-phenotype correlation in Chinese HS patients has been seldom reported (Qin L. et al., 2020). Here, we systematically studied the genetic and clinical characteristics of patients with HS in Hubei province, China, and we investigated the genotype-phenotype correlation in those patients.

## Materials and Methods

### Subjects

Twenty-three unrelated Chinese patients with suspected HS in Hubei province from Dec 2016 to Dec 2019 were included in this study. Patients were diagnosed according to the guidelines from the British Society for Hematology (Bolton-Maggs et al., 2012). This study was approved by Ethics Committee of Tongji Hospital, and written informed consent was obtained from patients or their legal guardians if under 18 years of age.

### Next-Generation Sequencing

Targeted NGS was performed as previously described (Wang X. et al., 2018). Genomic DNA was extracted from peripheral blood with the PANA9600 Automated Nucleic Acid Extraction System (Tianlong, Xi’an, China). DNA libraries were built using the Ampliseq Library Preparation Kit (Thermo Fisher, San Diego, United States) targeting the mentioned genes (*ANK1*, *SPTB*, *SLC4A1*, *SPTA1*, and *EPB42*). PCR products purified using AMPure XP beads (Beckman Coulter, Brea, CA, United States) were pooled together and amplified using the Ion PGM Hi-Q OT2 Kit on One-Touch Two (OT2) system, and they were further enriched on the ES machine and semiconductor sequencing was conducted with the Hi-Q Sequencing Kit using the Ion 316 or Ion 318 chip on the Ion Torrent Personal Genome Machine (PGM).

Raw data were aligned to the human hg19 reference genome sequence. Variant annotation was performed according to the Human Genome Variation Society (HGVS) recommendations. Annotated variants were filtered if the MAF > 0.01 in any of the following databases, including gnomAD^1^, ExAC^2^, and 1000G^3^. Filtered variants were further queried from the HGMD database^4^. Functional prediction of missense variants was performed using VarCards^5^ (Li et al., 2018). Splice site variants were predicted using the GENIE^6^, NetGene2^7^, and HSF3.1^8^ (Brunak et al., 1991; Hebsgaard et al., 1996; Desmet et al., 2009) programs. Identified variants were further confirmed using Sanger sequencing on an ABI 3500 Dx Genetic Analyzer (Thermo Fisher, San Diego, United States).

### Statistical Analysis

Patients were grouped into *ANK1* and *SPTB* groups based on mutated genes. Genotype-phenotype analysis was performed by comparing hemoglobin (Hb), red blood cells (RBC), mean corpuscular volume (MCV), mean corpuscular hemoglobin (MCH), and mean corpuscular hemoglobin concentration (MCHC) between different groups. Statistical analyses were performed using the GraphPad prism 5 software (GraphPad Software, Inc., San Diego, CA, United States). All data were analyzed using the Mann-Whitney *U*-test, and a *p*-value of < 0.05 was considered statistically significant.

## Results

### Clinical Features of Hereditary Spherocytosis Patients

The clinical features of these index patients were listed in Table 1. In summary, more than half of the patients (13/23) were less than 14 years old, and 14 out of 23 (60.9%) patients were female. Seven patients received splenectomy before their Hb and RBC levels recovered to normal, and the remaining sixteen patients were classified as moderate (Hb > 8.0 g/dL) to moderately severe (Hb: 6.0–8.0 g/dL). Eight of the moderate to moderately severe cases were aged ≤ 6 years old. The MCV, MCH, and MCHC levels were in the normal range for most patients. The Hb levels in patients before splenectomy were not available.

**TABLE 1:** Clinical and laboratory features of these included hereditary spherocytosis patients.

ID	Gender	Age	Hb	RBC	MCV	MCH	MCHC	Osmotic fragility	Spherocytosis	Splenomegaly	Splenectomy
1	Female	28 year	12.0	4.08	88.7	29.4	33.1	–	−	Yes	Yes
2	Male	31 year	–	–	–	–	–	–	–	Yes	Yes
3	Female	11 year	15.8	5.53	80.8 (↓)	28.6	35.3	–	Yes	Yes	Yes
4	Female	1 year	–	–	–	–	–	–	–	Yes	No
5	Male	4 year	–	–	–	–	–	–	–	Yes	No
6	Male	7 year	11.9	4.33	80.6 (↓)	27.5	34.1	↑	Yes	Yes	Yes
7	Male	24 year	12.3	3.82	90.3	32.2	35.7	↑	Yes	Yes	Yes
8	Male	14 year	15.9	5.35	84.9	29.7	35.0	↑	Yes	Yes	Yes
9	Female	23 year	8.4 (↓)	2.7 (↓)	84.8	31.1	36.7	–	Yes	Yes	No
10	Female	12 year	8.6 (↓)	2.89 (↓)	90.3	29.8	33.0	↑	Yes	Yes	No
11	Female	2 year	7.4 (↓)	2.87 (↓)	78 (↓)	25.8 (↓)	33.0	↑	Yes	Yes	No
12	Female	40 year	8.7 (↓)	2.83 (↓)	98.1	30.7	27.8 (↓)	↑	Yes	Yes	No
13	Female	15 year	7.8 (↓)	2.63 (↓)	90.9	29.7	32.6	↑	Yes	Yes	No
14	Male	4 year	–	–	–	–	–	–	–	Yes	No
15	Female	30 year	8.3 (↓)	2.51 (↓)	98.4	33.1	33.6	↑	Yes	Yes	No
16	Female	28 year	12.6	4.29	89.7	29.4	32.7	↑	Yes	Yes	Yes
17	Female	19 year	8.3 (↓)	2.62 (↓)	87.8	31.7	36.1	↑	Yes	Yes	No
18	Female	13 year	8.6 (↓)	2.79 (↓)	90	30.8	34.3	↑	Yes	Yes	No
19	Male	6 year	9.2 (↓)	3.4 (↓)	80.6 (↓)	27.1	33.6	–	−	Yes	No
20	Female	12 year	8.1 (↓)	2.59 (↓)	90.3	31.3	34.6	−	−	Yes	No
21	Male	3 year	8.8 (↓)	2.99 (↓)	86	29.4	34.2	↑	Yes	Yes	No
22	Male	6 year	8.2 (↓)	3.3 (↓)	74.8 (↓)	24.8 (↓)	33.2	−	Yes	Yes	No
23	Female	2 year	8.5 (↓)	3.25 (↓)	80.6 (↓)	26.2 (↓)	32.4	−	Yes	Yes	No

*Hb, hemoglobin; RBC, red blood cell; MCV, mean corpuscular volume; MCH, mean corpuscular hemoglobin; MCHC, mean corpuscular hemoglobin concentration. Hb (g/dL) ref: 11.5-15.0; RBC (10^12/L) ref: 3.8-5.1; MCV (fL) ref: 82.0-100.0; MCH (pg) ref: 27.0–34.0; MCHC (g/dL) ref: 31.6–35.4. Osmotic fragility ref (g/L): start NaCl concentration, 2.8–3.2, complete NaCl concentration, 4.2–4.6. The Hb levels before splenectomy of patients received splenectomy were not available.*

### Spectrum of Variants in Hereditary Spherocytosis Patients

Analysis of the targeted NGS panel data yielded causal variants in all 23 patients. Thirteen patients carried *ANK1* (NM_000037.3) variants, accounting for 57%. Ten patients had *SPTB* (NM_001024858.2) variants, accounting for 43% (Table 2). These variants included ten non-sense (45%), six indel (27%), four splice site (18%), one start-loss (5%), and one missense variant (5%).

**TABLE 2:** Variants detected in hereditary spherocytosispatients using next-generation sequencing (NM_000037.3 for *ANK1*, and NM_001024858.2 for *SPTB*).

Patient ID	Gene	Coding	Protein	Exon	Classification	Inheritance	Classification	Known/Novel
1	*ANK1*	c.856C > T	p.Arg286Ter	9	non-sense	Mother	Pathogenic	Known (Aggarwal et al., 2019)
2	*SPTB*	c.5799-2A > G	–	Intron 26	splice site	*De novo*	Pathogenic	Novel
3	*SPTB*	c.4211C > G	p.Ser1404Ter	19	non-sense	*De novo*	Pathogenic	Novel
4	*ANK1*	c.1800 + 1G > A	−	intron 16	splice site	Mother	Pathogenic	Novel
5	*ANK1*	c.2559-2A > C	−	Intron 23	splice site	Mother	Pathogenic	Novel
6	*ANK1*	c.701delT	p.Phe234SerfsTer19	7	indel	*De novo*	Pathogenic	Novel
7	*SPTB*	c.1310G > A	p.Trp437Ter	10	non-sense		Pathogenic	Novel
8	*ANK1*	c.2T > A	p.Met1Lys	1	start-loss		Uncertain significance	Novel
9	*ANK1*	c.1032_1034delGGC	p.Ala346del	10	indel	*De novo*	Pathogenic	Novel
10	*ANK1*	c.735delC	p.Ile245MetfsTer8	8	indel		Pathogenic	Novel
11	*ANK1*	c.4414C > T	p.Gln1472Ter	37	non-sense		Pathogenic	Novel
12	*ANK1*	c.2T > A	p.Met1Lys	1	start-loss		Uncertain significance	Novel
13	*ANK1*	c.3865delG	p.Glu1289LysfsTer16	32	indel		Pathogenic	Novel
14	*SPTB*	c.607A > T	p.Lys203Ter	5	non-sense	Father	Pathogenic	Novel
15	*SPTB*	c.4873C > T	p.Arg1625Ter	23	non-sense		Pathogenic	Known (Shen et al., 2019)
16	*SPTB*	c.2863C > T	p.Arg955Ter	15	non-sense		Pathogenic	Known (Aggarwal et al., 2019)
17	*ANK1*	c.341C > T	p.Pro114Leu	5	missense	*De novo*	Likely pathogenic	Known (van Vuren et al., 2019)
18	*SPTB*	c.563_566delCAGG	p.Gly189ThrfsTer22	4	indel	Father	Pathogenic	Novel
19	*SPTB*	c.3190C > T	p.Gln1064Ter	15	non-sense	Mother	Pathogenic	Novel
20	*SPTB*	c.5443G > T	p.Glu1815Ter	25	non-sense		Pathogenic	Novel
21	*ANK1*	c.2531_2532insT	p.Asp845GlyfsTer24	23	indel		Pathogenic	Novel
22	*SPTB*	c.2805-1G > T	−	Intron 14	splice site	*De novo*	Pathogenic	Novel
23	*ANK1*	c.4253G > A	p.Trp1418Ter	35	non-sense	Mother	Pathogenic	Novel

All these variants were heterozygous, and 18 out of 22 variants in our study were novel and absent from gnomAD, ExAC, 1000G, and HGMD. Most *ANK1* and *SPTB* variants were indel (5/12) and non-sense (7/10), respectively. The *ANK1* c.2T > A (p.Met1Lys) variant was identified in both patients 8 and 12. Patient 8 was a 14-year-old male who had received splenectomy, and patient 12 was a 40-year-old female. Family member analysis in thirteen families showed that six variants were *de novo*, accounting for 46%. All these variants were inherited in an AD pattern. The parents that resulted in heterozygous variants all showed hemolytic anemia and splenomegaly.

**FIGURE 1:**
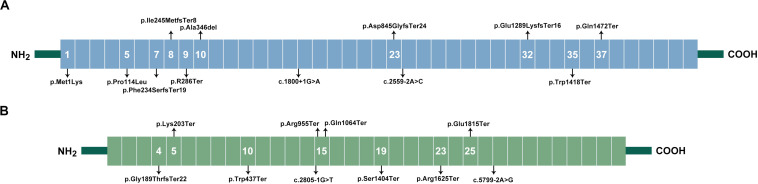
Spectrum of variants in HS patients. **(A)**, Locations of ankyrin. Ankyrin contained an 89 kDa domain, two ZU5 domains, a UPA domain, and a 55 kDa regulatory domain. The 89 kDa domain included 23 ANK repeats ranging from exon 1–21, which formed a spiral around a large central cavity involved in the binding of ion transporters. The two ZU5 and UPA domains, ranging from exon 26–33, forms a structural supramodule required for ankyrin’s function, and mediated interaction with β-spectrin. The 55-kDa domain, ranging from exon 34–42, included a death domain and was involved in the regulation of β-spectrin binding with protein band 3. The variants in *ANK1* gene were distributed in initial codon, ANK Repeats, UPA domain, and death domain. **(B)**, Location of β-spectrin. β-spectrin contains an actin-binding region (exon 1–7), 17 Spectrin repeats (exon 8–30), and a C-terminal. Variants in *SPTB* gene were mainly distributed in spectrin repeats, and two variants were in actin-binding region.

Ankyrin consists of a 24-tandem ankyrin repeat domain, a spectrin-binding domain, a death domain, and a regulatory C-terminal domain. Pathogenic variants were distributed in the first three domains and enriched in the 24-tandem ankyrin repeat domain, accounting for 75% (Figure 1A). In β-spectrin, deleterious variants were localized throughout the β-spectrin except for the tetramerization domain (Figure 1B).

### *In silico* Analysis of Missense and Splice Site Variants

*In silico* analysis of the missense variant (*ANK1* c.341C > T, p.Pro114Leu) by VarCards showed that 16 out of 19 algorithms showed a deleterious effect (Supplementary Table S1) and was highly conserved among species (Supplementary Table S2). This missense variant was found in a pair of twins, and it was *de novo*.

*In silico* analysis of the four splice site variants (*ANK1* c.1800 + 1G > A, *ANK1* c.2559-2A > C, *SPTB* c.2805-1G > T, and *SPTB* c.5799-2A > G) was performed using GENIE, NetGene2, and HSF 3.1. The splice score was decreased or the splice site was lost based on all three algorithms (Supplementary Table S3). We sequenced the reverse transcribed cDNA from peripheral blood in families carrying *ANK1* c.1800 + 1G > A and *SPTB* c.5799-2A > G, respectively. However, only normally spliced mRNA sequence was detected. Real time PCR and RNA sequencing may help to evaluate the expression and abnormally spliced mRNA.

### Genotype-Phenotype Correlation in Hereditary Spherocytosis Patients

**FIGURE 2:**
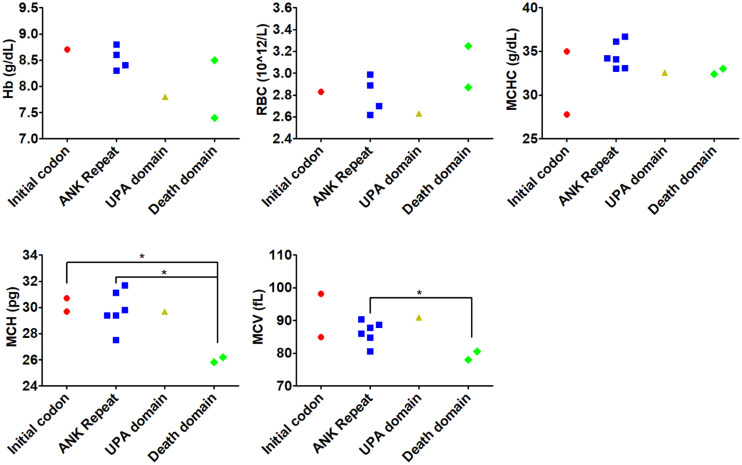
Genotype-phenotype correlation of variants in different domains within *ANK1*. Genotype-phenotype correlation was analyzed by comparing the indices including Hb, RBC, MCV, MCH, and MCHC in different domains within *ANK1*. *p < 0.05 compared with death domain group.

Genotype-phenotype correlation was analyzed by comparing the indices including Hb, RBC, MCV, MCH, and MCHC in different groups based on mutated genes. We found no significant difference for any indices between patients carrying *ANK1* or *SPTB* variants (Supplementary Table S4). However, ANK1 death domain variants tended to be associated with lower levels of MCV and MCH compared to other ANK1 domains (Figure 2).

Variable expressivities were observed. A pair of twins (number 17) carried the *de novo ANK1* missense *ANK1* c.341C > T (p.Pro114Leu) variant. The proband was admitted for cholelithiasis, anemia, and splenomegaly, but her twin sister did not suffer cholelithiasis. Moreover, patient 8 showed lower MCV and higher MCHC compared to patient 12, although they both harbored the heterozygous *ANK1* c.2T > A (p.Met1Lys) variant. In other analyzed families, affected family members showed similar phenotypes.

## Discussion

HS is the most common non−immune inherited hemolytic anemia, with variable expressivity (Iolascon et al., 2019), and the majority of HS was inherited in an AD manner. HS shows wide phenotypic and genotypic heterogeneity. The prevalence varies in different racial and ethnic regions, and the molecular spectrum differed in different regions. For example, the *ANK1* variant accounts for 40–65% in United States and Europe but 5–10% in Japan (Perrotta et al., 2008). Here, *ANK1* variants accounted for 57%, different to that observed in the Japanese population but similar to that in the Korean population (Park et al., 2016) In a cohort of 95 HS patients from the Netherlands, *SPTA1, ANK1*, and *SPTB* ranked as the top three genes with identified variants (van Vuren et al., 2019).

Here, Hb and RBC were both decreased in all patients without splenectomy. The MCV, MCH, and MCHC levels were all in the normal range, similar to a recent Indian study (Aggarwal et al., 2019). Moreover, 89% of the patients (17/19) showed MCHC < 35.9 g/dL, different from the findings reported in a study by Michaels et al., demonstrating that most HS cases had MCHC > 35.9 g/dL (Michaels et al., 1997). Our study further confirms that MCHC is less effective for determining HS.

NGS has promoted molecular HS diagnosis, including both targeted panel and WES (Roy et al., 2016; Russo et al., 2018; Xue et al., 2019; Qin L. et al., 2020). Here, the causal variants could be found in 100% of the cases using targeted NGS. In a recent retrospective study of children with HS by Tole et al. (2020), disease-causing variants had been identified in 160/166 (97%) children with HS. WES may help find the phenotype modifying genes (Aggarwal et al., 2019). Here, all variants were from *ANK1* and *SPTB* genes. Most *ANK1* and *SPTB* variants were indel (5/12) and non-sense (7/10), respectively. In the studies reported by Qin L et al. and Wang R et al., which included 35 Chinese patients from Tianjin of China and 38 patients from Beijing China, respectively, it was shown that *ANK1* and *SPTB* genes were the most frequently mutated genes (Wang R. et al., 2018; Qin L. et al., 2020). Qin L et al. found that six out of eight families showed a *de novo* variant, while we found that six out of thirteen families showed a lower rate of carrying *de novo* variants. Moreover, 21 out of 34 variants in the study by Qin et al. study were novel (Qin L. et al., 2020), and 18 out of 22 variants in our study were novel. These data indicate unique genetic characteristics of patients with HS in Hubei province, China.

Moreover, the laboratory indices showed unique clinical characteristics in Hubei province compared to Tianjin, China (Qin L. et al., 2020). We found no significant difference for any indices between patients carrying *ANK1* or *SPTB* variants. Our data were similar to the those reported in a study by Aggarwal et al. (2019) and Tole et al. (2020), where they found that the indices in patients with *ANK1* variants were similar to the *SPTB* group. Moreover, Tole et al. (2020) found that the variant type or location within each gene did not predict the disease severity. Here, ANK1 death domain variants were associated with lower levels of MCV and MCH compared to other ANK1 domains, but the number of patients was limited. MCV and MCH were much higher in the *ANK1* group than the *SPTB* group in the study by Qin L. et al. (2020). In a cohort of 25 Korean HS patients, anemia was most severe in the ANK1 spectrin-binding domain (Park et al., 2016). Similarly, van Vuren et al. found that variants affecting spectrin-binding of SPTA1, ANK1, and SPTB led to more severe phenotypes. They also found that red blood cell deformability measurements were associated with HS severity (van Vuren et al., 2019). Variable expressivity was observed in a pair of twins (number 17) with a *de novo ANK1* missense *ANK1* c.341C > T (p.Pro114Leu) variant. The proband was admitted for cholelithiasis, anemia, and splenomegaly, but her twin sister did not suffer cholelithiasis. The methylation level of the *ANK1* promoter region was correlated with *ANK1* expression (Smith et al., 2019). Variable expressivity observed in a pair of twins may be caused by the varied *ANK1* promoter region methylation level. Moreover, both patients 8 and 12 carried the heterozygous *ANK1* c.2T > A (p.Met1Lys) variant. They also showed different levels of MCV and MCHC, suggesting and confirming the variable expressivity. Taken together, these data indicate that both genetic and clinical characteristics of patients with HS may vary from different regions even of the same genetic background. However, the limited sample size may cause an inaccurate conclusion.

Sanger sequencing of the reverse transcribed cDNA from peripheral blood is a common and easy method to validate the effect of splice site variants on mRNA splicing (Wang X. et al., 2018). However, in this study, this method failed to detect abnormally spliced transcripts in two families carrying splice site variants. With the wide application of RNA sequencing, it may help to evaluate both the expression and abnormally spliced mRNA.

## Conclusion

In summary, we discovered 18 novel variants in the *ANK1* and *SPTB* genes from 23 Chinese patients with HS from Hubei province, central China. We found that NGS was an effective tool for rapid molecular diagnosis of HS. This is the first study to determine the genetic and clinical characteristics of patients with HS in Hubei Province, China.

## Data Availability Statement

The datasets generated for this study can be found in the SRA database with the following link: https://www.ncbi.nlm.nih.gov/sra/?term=PRJNA649395.

## Ethics Statement

The studies involving human participants were reviewed and approved by Ethics Committee of Tongji Hospital. Written informed consent to participate in this study was provided by the participants’ legal guardian/next of kin.

## Author Contributions

YL and LMC: project leads and designing the project. MH and LC: data collection. XW and YL: analysis. AZ and QH: data collection. XW: manuscript writing. All authors contributed to the article and approved the submitted version.

## Conflict of Interest

The authors declare that the research was conducted in the absence of any commercial or financial relationships that could be construed as a potential conflict of interest.

## Footnotes

1 http://gnomad.broadinstitute.org/

2 http://exac.broadinstitute.org/

3 http://asia.ensembl.org/index.html

4 http://www.hgmd.cf.ac.uk/ac/index.php

5 http://varcards.biols.ac.cn/

6 http://rulai.cshl.edu/new_alt_exon_db2/HTML/score.html

7 http://www.cbs.dtu.dk/services/NetGene2/

8 http://www.umd.be/HSF3/

## References

[B1] Aggarwal A. Jamwal M. Sharma P. Sachdeva M. U. S. Bansal D. Malhotra P. (2019). Deciphering molecular heterogeneity of Indian families with hereditary spherocytosis using targeted next-generation sequencing: first South Asian study. *Br. J. Haematol.* 188 784–795. 10.1111/bjh.16244 31602632

[B2] Bolton-Maggs P. H. Langer J. C. Iolascon A. Tittensor P. King M. J., and General Haematology Task Force of the British Committee for Standards in Haematology (2012). Guidelines for the diagnosis and management of hereditary spherocytosis–2011 update. *Br. J. Haematol.* 156 37–49. 10.1111/j.1365-2141.2011.08921.x 22055020

[B3] Brunak S. Engelbrecht J. Knudsen S. (1991). Prediction of human mRNA donor and acceptor sites from the DNA sequence. *J. Mol. Biol.* 220 49–65. 10.1016/0022-2836(91)90380-o 2067018

[B4] Desmet F. O. Hamroun D. Lalande M. Collod-Beroud G. Claustres M. Beroud C. (2009). Human splicing finder: an online bioinformatics tool to predict splicing signals. *Nucleic Acids Res.* 37:e67. 10.1093/nar/gkp215 19339519 PMC2685110

[B5] Di Resta C. Ferrari M. (2018). Next generation sequencing: from research area to clinical practice. *EJIFCC* 29 215–220. 30479607 PMC6247137

[B6] He B. J. Liao L. Deng Z. F. Tao Y. F. Xu Y. C. Lin F. Q. (2018). Molecular genetic mechanisms of hereditary spherocytosis: current perspectives. *Acta Haematol.* 139 60–66. 10.1159/000486229 29402830

[B7] Hebsgaard S. M. Korning P. G. Tolstrup N. Engelbrecht J. Rouze P. Brunak S. (1996). Splice site prediction in *Arabidopsis thaliana* pre-mRNA by combining local and global sequence information. *Nucleic Acids Res.* 24 3439–3452. 10.1093/nar/24.17.3439 8811101 PMC146109

[B8] Iolascon A. Andolfo I. Russo R. (2019). Advances in understanding the pathogenesis of red cell membrane disorders. *Br. J. Haematol.* 187 13–24. 10.1111/bjh.16126 31364155

[B9] Li J. Shi L. Zhang K. Zhang Y. Hu S. Zhao T. (2018). VarCards: an integrated genetic and clinical database for coding variants in the human genome. *Nucleic Acids Res.* 46 D1039–D1048. 10.1093/nar/gkx1039 29112736 PMC5753295

[B10] Michaels L. A. Cohen A. R. Zhao H. Raphael R. I. Manno C. S. (1997). Screening for hereditary spherocytosis by use of automated erythrocyte indexes. *J. Pediatr.* 130 957–960. 10.1016/s0022-3476(97)70283-x 9202619

[B11] Park J. Jeong D. C. Yoo J. Jang W. Chae H. Kim J. (2016). Mutational characteristics of ANK1 and SPTB genes in hereditary spherocytosis. *Clin. Genet.* 90 69–78. 10.1111/cge.12749 26830532

[B12] Perrotta S. Gallagher P. G. Mohandas N. (2008). Hereditary spherocytosis. *Lancet* 372 1411–1426. 10.1016/S0140-6736(08)61588-61583 18940465

[B13] Prodan Zitnik I. Cerne D. Mancini I. Simi L. Pazzagli M. Di Resta C. (2018). Personalized laboratory medicine: a patient-centered future approach. *Clin. Chem. Lab. Med.* 56 1981–1991. 10.1515/cclm-2018-2181 29990304

[B14] Qin L. Nie Y. Zhang H. Chen L. Zhang D. Lin Y. (2020). Identification of new mutations in patients with hereditary spherocytosis by next-generation sequencing. *J. Hum. Genet.* 65 427–434. 10.1038/s10038-020-0724-z 31980736

[B15] Roy N. B. Wilson E. A. Henderson S. Wray K. Babbs C. Okoli S. (2016). A novel 33-Gene targeted resequencing panel provides accurate, clinical-grade diagnosis and improves patient management for rare inherited anaemias. *Br. J. Haematol.* 175 318–330. 10.1111/bjh.14221 27432187 PMC5132128

[B16] Russo R. Andolfo I. Manna F. Gambale A. Marra R. Rosato B. E. (2018). Multi-gene panel testing improves diagnosis and management of patients with hereditary anemias. *Am. J. Hematol.* 93 672–682. 10.1002/ajh.25058 29396846

[B17] Shen H. Huang H. Luo K. Yi Y. Shi X. (2019). Two different pathogenic gene mutations coexisted in the same hereditary spherocytosis family manifested with heterogeneous phenotypes. *BMC Med. Genet.* 20:90 10.1186/s12881-019-0826-827 PMC6534931 31126250

[B18] Smith A. R. Smith R. G. Burrage J. Troakes C. Al-Sarraj S. Kalaria R. N. (2019). A cross-brain regions study of ANK1 DNA methylation in different neurodegenerative diseases. *Neurobiol. Aging* 74 70–76. 10.1016/j.neurobiolaging.2018.09.024 30439595

[B19] Tole S. Dhir P. Pugi J. Drury L. J. Butchart S. Fantauzzi M. (2020). Genotype-phenotype correlation in children with hereditary spherocytosis. *Br. J. Haematol.* 10.1111/bjh.16750 32436265

[B20] van Vuren A. van der Zwaag B. Huisjes R. Lak N. Bierings M. Gerritsen E. (2019). The complexity of genotype-phenotype correlations in hereditary spherocytosis: a cohort of 95 patients: genotype-phenotype correlation in hereditary spherocytosis. *Hemasphere* 3:e276. 10.1097/HS9.0000000000000276 31723846 PMC6745925

[B21] Wang C. Cui Y. Li Y. Liu X. Han J. (2015). A systematic review of hereditary *Spherocytosis* reported in Chinese biomedical journals from 1978 to 2013 and estimation of the prevalence of the disease using a disease model. *Intractable Rare Dis. Res.* 4 76–81. 10.5582/irdr.2015.01002 25984425 PMC4428190

[B22] Wang R. Yang S. Xu M. Huang J. Liu H. Gu W. (2018). Exome sequencing confirms molecular diagnoses in 38 Chinese families with hereditary spherocytosis. *Sci. China Life Sci.* 61 947–953. 10.1007/s11427-017-9232-9236 29572776

[B23] Wang X. Shen N. Huang M. Lu Y. Hu Q. (2018). Novel hereditary spherocytosis-associated splice site mutation in the ANK1 gene caused by parental gonosomal mosaicism. *Haematologica* 103 e219–e222. 10.3324/haematol.2017.186551 29449435 PMC5927983

[B24] Xue J. He Q. Xie X. Su A. Cao S. (2019). Clinical utility of targeted gene enrichment and sequencing technique in the diagnosis of adult hereditary spherocytosis. *Ann. Transl. Med.* 7:527. 10.21037/atm.2019.09.163 31807509 PMC6861754

[B25] Zou W. Zhang Z. Tan Y. Zhang L. (2020). Hereditary spherocytosis associated with gilbert syndrome diagnosed with liver biopsy examination and exome sequencing. *J. Coll. Phys. Surg. Pak.* 30 213–215. 10.29271/jp.2020.02.213 32036834

