# Supplementary material for: Genetic and Clinical Characteristics of Patients With Hereditary Spherocytosis in Hubei Province of China
Source: Front Genet. 2020 Aug 18;11:953. doi: 10.3389/fgene.2020.00953 (PMC7461774; doi:10.3389/fgene.2020.00953)
Supplement: Supplementary file 1 [file Data_Sheet_1.docx]

Table S1 In silico analysis of missense variant identified in this study

| **Algorithm** | **Score** | **Prediction** |
| --- | --- | --- |
| SIFT | 0.0 | Damaging |
| Polyphen-2_HDIV | 1.0 | Probably damaging |
| Polyphen-2_HVAR | 1.0 | Probably damaging |
| LRT | 0.0 | Deleterious |
| MutationTaster | 1.0 | Disease causing |
| MutationAssessor | 2.9 | Medium |
| FATHMM | -0.6 | Tolerable |
| PROVEAN | -8.4 | Damaging |
| VEST3 | 0.9 | Damaging |
| MetaSVM | 0.2 | Damaging |
| MetaLR | 0.6 | Damaging |
| M-CAP | 0.2 | Damaging |
| CADD | 32.0 | Damaging |
| DANN | 1.0 | Damaging |
| FATHMM_MKL | 1.0 | Damaging |
| Eigen | 0.9 | Damaging |
| GenoCanyon | 1.0 | Damaging |
| fitCons | 0.5 | Tolerable |
| REVEL | 0.8 | Damaging |
| ReVe | 1.0 | Damaging |
| ClinPred | 1.0 | Pathogenic |

Table S2 In silico analysis of conservation among species for missense variant identified in this study

| **Algorithm** | **Score** | **Prediction** |
| --- | --- | --- |
| GERP++ | 5.9 | Conserved |
| phyloP | 10.0 | Conserved |
| phastCons | 1.0 | Conserved |
| SiPhy | 20.3 | Conserved |

Table S3 In silico analysis of splice site variant identified in this study

| **Variant** | **GENIE** (wild type vs mutant) | **NetGene2** (wild type vs mutant) | **HSF 3.1** (wild type vs mutant) |
| --- | --- | --- | --- |
| *ANK1* c.1800+1G>A | 7.6 vs -3.1 | 0.95 vs - | 91.23 vs - |
| *ANK1* c.2559-2A>C | 6.4 vs -4.6 | 0.52 vs - | 87.57 vs - |
| *SPTB* c.2805-1G>T | 7.0 vs -4.0 | 0.34 vs - | 88.11 vs - |
| *SPTB* c.5799-2A>G | 11.2 vs 0.2 | 1.00 vs - | 91.7 vs - |

Note: - means no splice site was predicted.

Table S4 Genotype-phenotype correlations in HS patients

| **Test** | **Variants in *ANK1*** | **Variants in *SPTB*** | **p value** | **Reference** |
| --- | --- | --- | --- | --- |
| Hb | 8.31 ± 0.48 | 8.50 ± 0.44 | 0.54 | 11.5-15.0 g/dL |
| RBC | 2.85 ± 0.21 | 2.92 ± 0.41 | 0.69 | 3.8-5.1 10^12^/L |
| MCV | 86.43 ± 5.68 | 86.86 ± 7.52 | 0.89 | 82.0-100.0 fL |
| MCH | 29.18 ± 1.91 | 29.66 ± 2.77 | 0.66 | 27.0-34.0 pg |
| MCHC | 33.45 ± 2.35 | 34.13 ± 1.04 | 0.46 | 31.6-35.4 g/dL |

Note: Patients received splenectomy were not included in Hb and RBC analysis
